# Pre-operative clinical predictors for cardiology referral prior to total joint arthroplasty: the ‘asymptomatic’ patient

**DOI:** 10.1186/s13018-020-02042-5

**Published:** 2020-11-10

**Authors:** Yassin Elsiwy, Tristan Symonds, Kenji Doma, Kaushik Hazratwala, Matthew Wilkinson, Hayley Letson

**Affiliations:** 1 College of Medicine and Dentistry, James Cook University, 1 James Cook Drive, Townsville, QLD 4811 Australia; 2 Orthopaedic Research Institute of Queensland, 7 Turner Street, Townsville, QLD 4812 Australia; 3 College of Healthcare Sciences, James Cook University, 1 James Cook Drive, Townsville, QLD 4811 Australia; 4 School of Medicine, University of Tasmania, Medical Science Precinct, 17 Liverpool Street, Hobart, TAS 7000 Australia

**Keywords:** Cardiac, Arthroplasty, TKA, THA, Risk factor, Complication

## Abstract

**Background:**

No validated pre-operative cardiac risk stratification tool exists that is specific for total hip and total knee arthroplasty (THA and TKA, respectively). To reduce the risk of post-operative cardiac complication, surgeons need clear guidance on which patients are likely to benefit from pre-operative cardiac optimisation. This is particularly important for asymptomatic patients, where the need is harder to determine.

**Methods:**

Primary THA and TKA performed between January 1, 2010, and December 31, 2017, were identified from a single orthopaedic practice. Over 25 risk factors were evaluated as predictors for patients requiring additional cardiac investigation beyond an ECG and echocardiogram, and for cardiac abnormality detected upon additional investigation. A multivariate logistic regression was conducted using significant predictor variables identified from inferential statistics. A series of predictive scores were constructed and weighted to identify the influence of each variable on the ability to predict the detection of cardiac abnormality pre-operatively.

**Results:**

Three hundred seventy-four patients were eligible for inclusion. Increasing age (*p* < 0.001), history of cerebrovascular accident (*p* = 0.018), family history of cardiovascular disease (FHx of CVD) (*p* < 0.001) and decreased ejection fraction (EF) (*p* < 0.001) were significant predictors of additional cardiac investigation being required. Increasing age (*p* = 0.003), male gender (*p* = 0.042), FHx of CVD (*p* = 0.001) and a reduced EF (*p* < 0.001) were significantly predictive for the detection of cardiac abnormality upon additional cardiac investigation.

**Conclusions:**

Increasing age, male gender, FHx of CVD and decreased ejection fraction are important risk factors to consider for pre-operative cardiac optimisation in THA and TKA patients. These findings can be applied towards future predictive models, to determine which asymptomatic patients are likely to benefit from pre-operative cardiac referral.

## Background

Total joint arthroplasty (TJA) is the mainstay of treatment for end-stage osteoarthritis (OA) of the hip and knee, improving pain, function and quality of life [1]. Cardiac complication represents a major cause of morbidity and mortality after TJA and is associated with increased hospital mortality, length of stay and health expenditure [2–6]. Though rates of major cardiac complication are reported as low as 0.2–0.8%, this risk becomes increasingly significant given the projected 174 and 673% increase in total hip (THA) and total knee (TKA) arthroplasty, respectively, by 2030 in the USA [2, 7–13].

Previous studies have identified risk factors for post-operative cardiac complication in TKA and THA cohorts; however, a recent systematic literature review identified several limitations within the current literature [14]. Firstly, there is inconsistency regarding which risk factors are significant predictors of cardiac complication. Secondly, few studies extended their findings by way of a predictive model of multiple significant risk factors that can be adopted for cardiac risk stratification prior to TJA. Although Waterman and colleagues proposed one such model using age ≥ 80 years, history of cardiac disease, and hypertension, this tool has not been validated and further work is required to determine the value of other important risk factors in pre-operative cardiac risk stratification [3]. Therefore, no validated, orthopaedic-specific model(s) currently exist to facilitate pre-operative cardiac risk stratification. Although pre-operative referral for cardiac optimisation is clear-cut for the symptomatic patient already exhibiting signs of cardiovascular disease [15], the indication is less clear for asymptomatic patients not yet exhibiting clinical manifestations of a potential underlying and established cardiac disease.

The purpose of this study was to determine which risk factors should prompt orthopaedic surgeons to refer ‘asymptomatic’ patients for cardiac optimisation prior to TKA or THA. The primary objective being to identify risk factors which can be indicative of underlying and undiagnosed cardiac disease in an asymptomatic patient, which may otherwise place the patient at risk of cardiac complication peri- or post-TJA. Based on prior analysis, we hypothesised that age and history of cardiac disease will be significant predictors. However, we also sought to further determine the predictive value of additional potentially important risk factors [14]. Significant risk factors identified in this study may contribute to the development of a more comprehensive cardiac risk stratification tool that may better guide pre-operative cardiac referral for the ‘asymptomatic’ TJA patient.

## Methods

This study was approved by the Mater Health Services North Queensland Human Research Ethics Committee (Approval: MHS20180424-01). The study was a retrospective consecutive short series of data collected between January 1, 2010, and December 30, 2017, at a single orthopaedic practice in Australia. Patients eligible for inclusion were those who underwent a TKA or THA and received pre-operative cardiology assessment including an electrocardiogram (ECG) and echocardiogram (ECHO) as a baseline investigation. Pre-operative cardiology assessment was standard practice for all patients, regardless of suspected risk, for this institution. Surgery was performed by a single surgeon and pre-operative cardiology review was completed by one of seven cardiologists. Patients who underwent bilateral TKA or THA, revision arthroplasty, or had a surgical indication other than osteoarthritis were excluded. As this study aims to risk-stratify within an asymptomatic TJA population, patients identified to have symptomatic cardiac disease including angina, evidence of heart failure (New York Heart Association class > 1), orthopnoea, paroxysmal nocturnal dyspnoea, and/or peripheral oedema, at the initial surgical consultation, were excluded. A flowchart of the inclusion and exclusion criteria is provided as Supplementary Figure 1.

Data was collected by two investigators from electronic records. Variables were selected based on a prior systematic review examining risk factors for cardiac complication after TKA and THA [14]. The predictor variables included patient demographics; medical history (cardiac and non-cardiac); family history of cardiac disease (FHx of CVD), defined as history of myocardial infarction, cardiac arrest, sudden cardiac death, ischaemic heart disease, coronary artery disease or congestive heart failure in a first-degree relative; and results of baseline cardiac investigation (ECG and ECHO). Data extracted were age, gender, body mass index (BMI), diabetes, hypertension (HTN), hypercholesterolaemia, smoking history, alcohol history, chronic kidney disease, chronic obstructive pulmonary disease, asthma, obstructive sleep apnoea, chronic heart failure, myocardial infarction, coronary artery disease (CAD), valvular disease, arrhythmia, peripheral vascular disease, cerebrovascular accident (CVA) and venous thromboembolism [14]. The outcome variables for this analysis were (1) additional cardiac investigation (ACI) ordered by cardiologist (e.g. angiogram, CT-coronary angiography (CTCA), stress test, or myocardial perfusion scan (MIBI)) and (2) abnormality detected upon additional cardiac investigation (ADACI). Post-operative cardiac complications (defined as cardiac event within 30 days), as well as surgical delays (defined as a change in the date of surgery for cardiac optimisation), and surgical cancellation by cardiologist, were also recorded.

### Statistical methodology

Data analysis was performed using SPSS 24.0 (IBM) with the alpha level set at 0.05. The measure of central tendency and dispersion was reported as mean ± standard deviation, and prevalence as frequencies. Based on Shapiro-Wilk test, continuous parameters were log-transformed prior to inferential statistical analyses. The discriminant and predictive capacities for ACI and ADACI were assessed using a three-tiered approach (Supplementary Figure 2). Firstly, an independent *t* test was used to determine differences in age, BMI and ejection fraction (EF) between patients with, and without, the need for ACI, and between patients with, and without, ADACI. Similar inter-individual comparisons were conducted for all other nominal variables using a chi-squared test of independence. Crude odds ratio and associated 95% confidence interval (CI) of all continuous and nominal variables were calculated using MedCalc (Version 19.0.7, Belgium). Secondly, a stepwise, multivariate logistic regression was conducted to determine whether the variables that were incorporated in the inferential statistics (i.e. independent variables) predicted ACI and ADACI (i.e. dependent variables). Adjusted odds ratio and associated 95% CI derived from the multivariate logistic regression were also reported. According to the Hosmer and Lemeshow test, the dependent variables were not significant (*p* > 0.05), demonstrating appropriate goodness of fit for the multivariable logistic regression, and that the sample size provided sufficient predictive capacity for the model. Finally, the variance inflation factor of both multivariate logistic regression models were < 5 for the independent variables, indicating that the predictors demonstrated minimal multi-collinearity [16].

### Internal validation of significant predictor variables for ADACI

Based on the performance of predictor variables in the multivariate logistic regression model, a series of predictive scores were constructed that weighted the influence of each predictor on the odds of a cardiac abnormality being detected upon additional cardiac investigation. Numerical variables were normalised against the mean of the cohort, while categorical variables were allocated an arbitrary value of 1 for presence of the risk factor and 0 for its absence. To determine the discriminant capacity for ADACI, receiver operator characteristics (ROC) curves were generated to compute the area under the curve or c-statistic (c-statistic; 95% CI). The model weighted predictors based on the odds of an abnormality detected upon additional cardiac investigation to closely reflect each risk factors’ weight in the predictive model. Risk factors with an odds ratio 1 ≤ OR < 2 were multiplied by 1 point, 2 ≤ OR < 3 multiplied by 2 points, and ≥ 3 multiplied by 3 points to produce a final score. The discriminative capability of the model was evaluated using the c-statistic. The stepwise, multivariate logistic regression allowed selection of pertinent predictors for the ROC curves. Appropriate model fitting of these explanatory variables were assessed via Hosmer-Lemeshow goodness of fit test, in conjunction with the likelihood-ratio chi-squared test. Accordingly, the results of the tests were not statistically significant, indicating that the statistical model was well calibrated, and our model improved from the null model. Furthermore, the Akaike information criterion was examined across several iterations as part of the stepwise process, with the model exhibiting the smallest value selected to ensure a better-fit model. The assumption of linearity of the multivariate logistic regression between the log odds and predictor variables was assessed using the Box-Tidwell test in Stata (version 16, Texas). With regard to the ROC curves, the cut-off demonstrating the greatest sensitivity and specificity was chosen.

## Results

### Cohort composition and patient characteristics

A total of 374 patients were eligible for inclusion. Four hundred forty-three patients were directly identified based on the inclusion criteria, though THA and TKA are not the only procedures performed at this centre. From a total of 443 patients, 7 patients were excluded due to cancellation of surgery for non-medical reasons and 62 patients were excluded due to missing crucial data, namely ECG and ECHO reports, which were unable to be retrieved from medical records (Supplementary Figure 1). Of the 374 patients eligible for inclusion, 272 (72.7%) underwent TKA, and 102 (27.3%) underwent THA (Additional Table 1). The mean age of the cohort was 69.9 ± 9.0 years, and the majority of patients were male (54.0%). Hypertension was the most common risk factor (65.2%), followed by hypercholesterolaemia (57.8%) and diabetes (15.0%) (Additional Table 1). More than one third (35.3%) of patients reported a previous cardiac history, and 19.3% reported a FHx of CVD. There were 22 (5.9%) current smokers and 91 (24.3%) ex-smokers in this population. The mean EF was 63.82 ± 8.28%, and an ECG abnormality was found in 115 patients (30.7%) during pre-operative cardiology review (Additional Table 1).

In the total population, six patients did not proceed with surgery following cardiology review: one had severe valvular disease identified on history, one had ECG evidence of a prior infarction not previously known, two had evidence of severe stenosis on CTCA, and three were cancelled due to non-cardiac reasons. Post-operative arrhythmia (atrial fibrillation) was reported in seven patients, with no other cardiac complications post-operatively.

### Additional cardiac investigation (ACI)

One hundred thirty-eight patients underwent ACI following pre-operative cardiology review of ECG/ECHO. The mean age was 72.3 ± 8.7 years, and age was found to be significantly associated with ACI (*p* < 0.001) (Table 1). Hypertension, previous cardiac history and FHx of cardiac disease were more prevalent in this cohort compared to the total patient population, and these variables were all significant for additional cardiac investigation (*p* = 0.001, *p* = 0.001 and *p* < 0.001, respectively) (Table 1). More specifically, a history of CAD or CVA was significantly associated with ACI (*p* = 0.004 and *p* < 0.001, respectively). An ECG abnormality, such as heart block, atrial fibrillation, evidence of prior ischaemic events, or ectopic beats, was detected in 38.4% patients that went on to have additional cardiac investigation (*p* = 0.010). The mean EF was similar to the mean for the total patient population (62.0% vs. 63.8%), and EF was significantly associated with ACI (*p* = 0.005) (Table 1).

**Table 1:** Risk factors associated with additional cardiac investigation

Variable	ACI		Statistics					
	Yes (*n* = 138)	No (*n* = 236)	*χ*^2^	*p* value	*z* score	Crude OR	95% CI	*p* value
*Demographics*								
Age (years)	72.3 ± 8.7	68.5 ± 8.9	–	< 0.001	–	1.05	1.03–1.08	< 0.001
Male	81 (58.7%)	121 (51.3%)	1.93	0.164	1.4	1.35	0.88–2.06	0.170
Female	57 (41.3%)	115 (48.7%)	1.93	0.164	-1.4	0.74	0.48–1.13	0.170
BMI	31.4 ± 5.5	31.1 ± 5.3	–	0.592	–	1.01	0.97–1.05	0.590
*Past medical Hx*								
Diabetes	27 (19.6%)	29 (12.3%)	3.62	0.057	1.9	1.74	0.98–3.08	0.060
Hypertension	105 (76.1%)	139 (58.9%)	11.35	0.001	3.4	2.22	1.39–3.55	<0.001
Hypercholesterolaemia	88 (63.8%)	128 (54.2%)	3.24	0.072	1.8	1.49	0.96–2.29	0.070
Smoking	11 (8.0%)	11 (4.7%)	5.97	0.050	1.3	2.03	0.85–4.88	0.110
Ex-smoker	41 (29.7%)	50 (21.2%)	5.97	0.050	1.9	1.67	1.03–2.7	0.040
Alcohol	13 (9.4%)	29 (12.3%)	0.72	0.397	- 0.8	0.74	0.37–1.48	0.400
CKD	12 (8.7%)	11 (4.7%)	2.47	0.292	1.6	1.95	0.83–4.54	0.120
Asthma	16 (11.6%)	28 (11.9%)	0.006	0.938	-0.1	0.97	0.51–1.87	0.940
COPD	7 (5.1%)	7 (3.0%)	1.07	0.300	1.0	1.75	0.60–5.09	1.000
OSA	13 (9.4%)	3 (12.7%)	0.93	0.336	-1.0	0.71	0.36–1.42	0.340
*Past CV Hx*								
Cardiac Hx total	63 (45.7%)	69 (29.2%)	10.27	0.001	3.2	2.03	1.31–3.15	0.002
CCF	3 (2.2%)	5 (2.1%)	0.001	0.972	0.0	1.03	0.24–4.36	0.970
MI	14 (10.1%)	14 (5.9%)	2.23	0.135	1.5	1.79	0.83–3.88	0.140
CAD	38 (27.5%)	36 (15.3%)	8.28	0.004	2.9	2.11	1.26–3.53	0.005
Valvular disease	10 (7.2%)	15 (6.4%)	0.11	0.739	0.3	1.15	0.50–2.64	0.740
Arrhythmia	25 (18.1%)	30 (12.7%)	2.04	0.154	1.4	1.52	0.85–2.71	0.160
PVD	4 (2.9%)	3 (1.3%)	1.26	0.262	1.1	2.32	0.51 –10.52	0.280
CVA	19 (13.8%)	8 (3.4%)	14.00	<0.001	3.7	4.55	1.93 –10.70	<0.001
VTE	8 (5.8%)	6 (2.5%)	2.56	0.110	1.6	2.36	0.80–6.95	0.120
*Family Hx of cardiac disease*	42 (30.4%)	30 (12.7%)	17.60	<0.001	4.2	3.00	1.77–5.09	<0.0001
*Baseline cardiac Ix*								
ECG abnormality	53 (38.4%)	62 (26.3%)	6.02	0.014	2.5	1.75	1.12–2.74	0.001
Ejection fraction (%)	62.0 ± 10.2	64.9 ± 6.7	–	0.005	–	0.96	0.93–0.99	0.002

Numerical predictor variables (age, BMI, ejection fraction) are presented as mean ± standard deviation and were assessed using an independent *t* test. Categorical predictor variables are presented as frequencies and were assessed using chi-squared test of independence. The crude ratio and associated 95% CI are also reported. Significant predictor variables are indicated by *p* < 0.05

*ACI* additional cardiac investigation, *OR* odds ratio, *BMI* body mass index, *CV* cardiovascular, *Hx* history, *CCF* chronic heart failure, *MI* myocardial infarction, *CAD* coronary artery disease, *PVD* peripheral vascular disease, *CVA* cerebrovascular accident, *VTE* venous thromboembolism, *CKD* chronic kidney disease, *COPD* chronic obstructive pulmonary disease, *OSA* obstructive sleep apnoea, *ECG* electrocardiogram, *Ix* investigation

According to the logistic regression, age (OR 1.06, 95% CI 1.03–1.09, *p* < 0.001), CVA (OR 3.83, 95% CI 1.26–11.61, *p* = 0.018) and FHx of CVD (OR 4.20, 95% CI 2.27–7.80, *p*<0.001) significantly predicted the need for ACI, while increased EF was predictive of cardiologist not referring for further investigation (OR 0.94, 95% CI 0.91–0.97, *p* < 0.001) (Table 2). Hypertension and coronary artery disease increased the likelihood of requiring ACI 1.63-fold and 1.67-fold, respectively; however, these risk factors were not significant predictors (*p* = 0.080 and *p* = 0.106, respectively).

**Table 2:** Multivariate logistic regression for additional cardiac investigation

					95% Cl	
Predictors	*β*	S.E	*p*	OR	Low	High
*Demographics*						
Age	0.06	0.02	< 0.001	1.06	1.03	1.09
HTN	0.49	0.28	0.080	1.63	0.94	2.83
*Cardiac history*						
CAD	0.51	0.32	0.106	1.67	0.90	3.12
CVA	1.34	0.57	0.018	3.83	1.26	11.61
FHx of CVD	1.44	0.32	< 0.001	4.20	2.27	7.80
*Baseline cardiac Ix*						
ECG abnormality	0.29	0.28	0.309	1.34	0.77	2.33
Ejection fraction	− 0.06	0.02	< 0.001	0.94	0.91	0.97

Multivariate logistic regression results with adjusted odds ratios (OR) and associated 95% confidence intervals (Cl). Significant predictor variables are indicated by *p* < 0.05

*HTN* hypertension, *CAD* coronary artery disease, *CVA* cerebrovascular accident, *FHx* family history, *CVD* cardiovascular disease, *Ix* investigation, *ECG* electrocardiogram

### Abnormality detected upon additional cardiac investigation (ADACI)

Fifty patients were found to have an abnormality upon additional cardiac investigation. The mean age was 73.3 ± 8.4 years, and age was found to be significantly associated with the incidence of an abnormal cardiac finding (*p* =0 .004) (Table 3). Male gender was more prevalent compared with the total cohort (70% vs. 54%) and was significantly associated with ADACI (*p* = 0.015), as were current and ex-smokers (both *p* = 0.009) (Table 3). In contrast to the ACI group, cardiac history was not significantly associated with detection of an abnormality on additional cardiac investigation (*p* = 0.601). However, CVA and FHx of CVD were significant (*p* = 0.010 and *p* = 0.014, respectively). ECG abnormality (*p* = 0.005) and EF (*p* < 0.001) from baseline cardiac assessment were also both significantly associated with ADACI (Table 3).

**Table 3:** Risk factors associated with abnormality detected upon additional cardiac investigation

	ADACI		Statistics					
	Yes (*n* = 50)	No (*n* = 324)	*χ*^2^	*p* value	*z* score	Crude OR	95% CI	*p* value
*Demographics*								
Age (years)	73.3 ± 8.4	69.4 ± 9.0	-	0.004	-	1.05	1.02–1.09	0.005
Male	35 (70%)	167 (51.5%)	5.94	0.015	2.4	2.19	1.15–4.17	0.020
Female	15 (30%)	157 (48.5%)	5.94	0.015	-2.4	0.46	0.24–0.87	0.020
BMI	31.4 ± 5.9	31.2 ± 5.3	-	0.880	-	1.01	0.95–1.06	0.870
*Past medical Hx*								
Diabetes	9 (18%)	47 (14.5%)	0.42	0.519	0.6	1.30	0.59–2.84	0.520
Hypertension	37 (74%)	207 (63.9%)	1.95	0.162	1.4	1.61	0.82–3.15	0.170
Hypercholesterolaemia	30 (60.0%)	186 (57.4%)	0.12	0.730	0.3	1.11	0.61–2.04	0.730
Smoking	6 (12.0%)	16 (4.9%)	9.52	0.009	2.0	3.39	1.22–9.42	0.020
Ex-smoker	18 (36.0%)	73 (22.5%)	9.52	0.009	2.1	2.23	1.16–4.29	0.020
Alcohol	6 (12.0%)	36 (11.1%)	0.03	0.853	0.2	1.09	0.43–2.74	0.850
Asthma	7 (14.0%)	37 (11.4%)	0.28	0.598	0.5	1.26	0.53–3.01	0.600
COPD	2 (4.0%)	12 (3.7%)	0.01	0.918	0.1	1.08	0.24–4.99	0.920
OSA	4 (8.0%)	39 (12.0%)	0.69	0.405	-0.8	0.64	0.22–1.86	0.410
*Past CV Hx*								
Cardiac Hx total	16 (32%)	116 (35.8%)	0.27	0.601	-0.5	0.84	0.45–1.59	0.600
CCF	0 (0.0%)	8 (2.5%)	1.26	0.261	-1.1	0.37	0.02–6.49	0.500
MI	6 (12.0%)	22 (6.8%)	1.70	0.193	1.3	1.87	0.72–4.87	0.200
CAD	11 (22%)	63 (19.4%)	0.18	0.673	0.4	1.14	0.56–2.34	0.710
Valvular disease	5 (10.0%)	20 (6.2%)	1.02	0.313	1.0	1.69	0.60–4.73	0.320
Arrhythmia	6 (12.0%)	49 (15.1%)	0.34	0.562	-0.6	0.79	0.32–1.94	0.610
PVD	2 (4.0%)	5 (1.5%)	1.42	0.233	1.2	2.66	0.50–14.09	0.250
CVA	8 (16.0%)	19 (5.9%)	6.64	0.010	2.6	2.45	1.04–5.74	0.040
VTE	4 (8.0%)	10 (3.1%)	2.90	0.088	1.7	2.73	0.82–9.07	0.100
CKD	3 (6.0%)	20 (6.2%)	0.47	0.790	0.0	0.97	0.28–3.37	0.970
*Family Hx of cardiac disease*	16 (32.0%)	56 (17.3%)	6.03	0.014	2.5	2.25	1.16–4.36	0.020
*Baseline cardiac Ix*								
ECG abnormality	24 (48.0%)	91 (28.1%)	8.07	0.005	2.8	2.36	1.29–4.33	0.005
Ejection fraction (%)	58.9 ± 11.6	64.6 ± 7.3	-	0.002	-	0.93	0.89–0.96	<0.001

Numerical predictor variables (age, BMI, ejection fraction) are presented as mean ± standard deviation and were assessed using an independent *t* test. Categorical predictor variables are presented as frequencies and were assessed using chi-squared test of independence. The crude ratio and associated 95% CI are also reported. Significant predictor variables are indicated by *p* < 0.05

*ADACI* abnormality detected upon additional cardiac investigation, *OR* odds ratio, *BMI* body mass index, *CV* cardiovascular, *Hx* history, *CCF* chronic heart failure, *MI* myocardial infarction, *CAD* coronary artery disease, *PVD* peripheral vascular disease, *CVA* cerebrovascular accident, *VTE* venous thromboembolism, *CKD* chronic kidney disease, *COPD* chronic obstructive pulmonary disease, *OSA* obstructive sleep apnoea, *Ix* investigation, *ECG* electrocardiogram

A total of 17 patients in the ADACI group had a surgical delay by the cardiologist to undergo pre-operative cardiac optimisation, whereas the remaining patients were able to be optimised without surgical delay. In the delayed group, 12 had an ECG abnormality, either atrial fibrillation or heart block, and five had an EF < 50% at baseline. Consequently, 15 had an abnormal angiogram and two had an abnormal MIBI.

Logistic regression revealed increased age, male gender, FHx of CVD, and reduced EF significantly predicted detection of a cardiac abnormality pre-operatively (Table 4). For every increased year in age, there was a significant 7% increase in ADACI (OR 1.07; 1.02–1.12 95% Cl, *p* = 0.003), while every percentage increase in EF reduced the likelihood of finding an abnormality by 7% (OR 0.93; 0.89–0.97 95% Cl, *p* < 0.001). Male gender was also a significant predictor for ADACI (OR 2.22; 1.03–4.79 95% Cl, *p* = 0.042). FHx of CVD was a strong predictor and significantly increased the likelihood of finding a cardiac abnormality 3.6-fold (OR 3.61; 1.65–7.89 95% CI, *p* = 0.001). Smoking status and history of CVA did not demonstrate any significant predictive capacity (Table 4).

**Table 4:** Multivariate logistic regression for abnormality detected upon additional cardiac investigation

					95% Cl	
Predictors	β	S.E	p	OR	Low	High
*Demographics*						
Age	0.07	0.02	0.003	1.07	1.02	1.12
Gender	0.80	0.39	0.042	2.22	1.03	4.79
Current smoker	0.80	0.67	0.231	2.22	0.60	8.17
Ex-smoker	− 0.46	0.40	0.250	0.63	0.29	1.38
*Cardiac history*						
CVA	1.01	0.59	0.087	2.74	0.86	8.71
*Family history*						
FHx of CVD	1.28	0.40	0.001	3.61	1.65	7.89
*Baseline cardiac Ix*						
ECG abnormality	0.23	0.39	0.556	1.26	0.59	2.68
Ejection fraction	− 0.08	0.02	< 0.001	0.93	0.89	0.97

Multivariate logistic regression results for abnormality detected upon additional cardiac investigation with adjusted odds ratios (OR) and associated 95% confidence intervals (Cl). Significant predictor variables are indicated by *p* < 0.05

*CVA* cerebrovascular accident, *FHx* family history, *CVD* cardiovascular disease, *Ix* investigation, *ECG* electrocardiogram

To further validate these findings, significant predictors for ADACI were evaluated in a predictive model using ROC curve analysis. EF and Age were normalised against the mean of the cohort, with a Mean EF of 63.8% and a Mean Age of 69.9 years. As outlined in the “Methods” section, the model weighted the influence of the predictor variables based on the odds ratio produced by the logistic regression, as follows: (2* Gender) + (3* FHx of CVD) + (Mean EF/Patient EF) + (Age/Mean Age). This model produced a cut-off score of 3.99 and maintained reasonable predictive capacity (c-statistic = 0.71), with 70% sensitivity and 65% specificity, and good calibration (*p* > 0.05).

## Discussion

Cardiovascular complication continues to represent up to 20% of all major post-operative complication following TJA [12, 14] and will become increasingly important with an ageing, increasingly obese, and more medically complex arthroplasty population [12, 17, 18]. Pre-operative cardiology optimisation is essential to reduce post-operative cardiac events. However, no validated, orthopaedic-specific cardiac risk stratification tools are available to guide orthopaedic referral of patients to cardiology [3, 19–21]. There has been no previous research on cardiac referral for the asymptomatic preoperative TJA population in which cardiac disease not yet exhibiting clinical manifestation but capable of contributing to risk of postoperative cardiac complication may exist. Over-referral is unsustainable [4, 22–24] and may unnecessarily delay surgery [25, 26]; however under-referral may lead to missed pre-operative optimisation for asymptomatic patients who otherwise would have benefited, and may contribute to post-operative cardiac complication.

The need for a pre-operative risk stratification tool for lower limb arthroplasty was recognised by Waterman et al., who proposed the TJA Cardiac Risk Index Score [3]. Though the results of this study are promising, their use of the National Surgical Quality Improvement Program (NSQIP) database limited their analysis as only risk factors recorded in the database could be evaluated, and patient follow-up was limited to only 30 days post-operative [3, 27]. Improved cohort design and broader evaluation of risk factors is required to determine all risk factors that should guide pre-operative cardiology referral. Risk factors identified by the recent review by Elsiwy et al. were evaluated in this series and tested for their capacity to predict the need for additional cardiac investigation pre-operatively and abnormality detected upon additional cardiac investigation. Uniquely in this study, all patients, including asymptomatic patients without known cardiac disease, were referred to cardiology pre-operatively for ECG/ECHO, thereby avoiding the bias of selective referral of only higher-risk patients. Furthermore, in contrast to all previous literature, risk factors in this study were not measured against their capacity to predict post-operative cardiac events, but rather the ability to predict cardiologist referral for additional investigation pre-operatively, and cardiac abnormalities detected during additional investigation, which may prompt cardiac optimisation and/or surgical delay. The additional advantage of examining these outcome variables in a population that has all been referred to cardiology is that the overall cardiovascular profile of an asymptomatic population can be determined, patients with silent disease can be identified and risk factors which can better guide pre-operative cardiac referral can be determined.

Importantly, our cohort had a demographic profile comparable to previous arthroplasty populations described in the literature. The mean age and the cardiac complication rate (1.87%) was similar to the Waterman study [3, 14], while the cardiovascular risk profile of our cohort was similar to that described by Łęgosz et al. in their analysis of cardiovascular risk of arthroplasty populations [4]. No patients in our series suffered a major cardiac event or post-operative mortality, reflecting the experience and expertise of the orthopaedic surgeon. Given the small cohort size of 374 patients, this is also consistent with the low rates of mortality (0.18%) and incidence of major cardiac complication (myocardial infarction or cardiac arrest) previously reported (0.2%) [2, 3], but may also be due to the fact that all patients were referred for cardiology review and optimisation pre-operatively. The importance of this is further demonstrated by the finding of ischaemic lesions during ACI in 17 ‘asymptomatic’ patients that went on to have surgical delays.

By assessing predictors of additional cardiac investigation, this study highlights for the first time the risk factors that cardiologists are more likely to utilise for pre-operative risk stratification of TKA and THA patients. In this series, cardiologists were more likely to conduct additional cardiac investigation based on increasing age, FHx of CVD, previous CVA, and reduced EF. Of interest, although CVA was considered important by the cardiologists, it did not show a significant predictive capacity for detecting cardiac abnormality. The link between CVA and post-operative cardiac complication is controversial [14]; however, these results support previous literature in which CVA was not significantly associated with post-operative cardiac complication [2, 7]. In contrast, reduced EF was a consistent predictor for both ACI and ADACI, though current literature does not support the use of routine echocardiogram in pre-operative cardiovascular screening [28, 29].

For cardiac risk stratification, the detection of a cardiac abnormality is most relevant, and these patients are most likely to benefit from pre-operative cardiac optimisation since they have demonstrated cardiac disease. Similar to multiple studies showing a strong association between increasing age and post-THA and TKA cardiac complication [2, 3, 7, 10–12], our analysis demonstrated a significant predictive capacity of every year of increase in age for the detection of a cardiac abnormality pre-operatively. Male gender was also a significant predictor for ADACI and should therefore be considered in pre-operative risk stratification. Interestingly, this was not reported by Waterman et al., although the influence of male gender on increasing the risk of cardiovascular complication is well established in the literature [10, 13]. A 3.6-fold increased risk of detecting a cardiac abnormality with a FHx of CVD suggests that this risk factor should also be considered in pre-operative cardiac risk stratification. Notably, this risk factor has not been considered in this context in previous literature and was not addressed in the review by Elsiwy et al. [14]. Although family history is a long-established risk factor for cardiovascular disease, perhaps it has not been considered in previous arthroplasty studies due to its non-modifiable nature [30]. In contrast to having a family history, patient cardiac history was not a significant predictor for ADACI. While an association between previous cardiac history and post-operative cardiac complication has been reported [2, 3, 7, 11, 12, 31], it is possible that patients in the current study were previously optimised so the influence of their cardiac history had been mitigated or reversed. This is also potentially true of patients with diabetes, hypertension, hypercholesterolaemia and other risk factors linked to cardiac complication post-TJA in the literature that did not show significance in our series.

These findings that increased age, male gender, FHx of CVD and decreased ejection fraction predicted cardiac abnormality were internally validated in a predictive model that produced a statistically significant c-statistic > 0.70 which was equivalent to the model proposed by Waterman [3], and had reasonable discriminative capacity and no evidence of statistically significant lack of fit (*p* > 0.05) [32]. The proposed model demonstrates an innovative method of producing an algorithm that can be used to risk-stratify TJA patients and guide pre-operative cardiac referral. In contrast to the methods previously described by Waterman et al. [3] and several other surgical risk calculators [19, 21, 33] where various iterations are tested until the highest c-statistic is obtained, risk factors included in the our model were weighted based on their odds ratios, taking into consideration the individual influence of each risk factor.

## Limitations

The authors acknowledge several limitations that prevent translation of the proposed risk stratification model into clinical practice at this time. Due to the small population, additional risk factors that occur in low incidence within this population were unable to be accurately investigated, and continuous variables age and EF were not able to be stratified to evaluate specific ranges. Small sample size may also explain why being a previous or current smoker showed significant association but did not achieve significant predictive capacity in the regression model, in contrast to current literature [14]. Furthermore, some parameters such as the American Society of Anesthesiologists (ASA) score, which has previously shown significance in the literature, were not available in this data set [3]. However, this study avoids the inherent limitations of larger database-driven studies already discussed and provides a unique cohort of asymptomatic patients, who were all referred to cardiology prior to TKA or THA. Another limitation is the inclusion of EF as a predictive risk factor. Since routine echocardiography is not currently evidence-based practice [29], not all patients will have this measurement readily available for pre-operative risk stratification. There is evidence, however, that echocardiography has a role in pre-operative evaluation of higher risk patients [29]. Given the results of the current study which suggest that even asymptomatic TJA patients may be high risk if they are over 70, male and have a family history of cardiovascular disease, the benefit of echocardiography in this subset of patients needs further evaluation. While the bias of cardiac referral has been removed in this study, there is still potential bias in the provision of additional cardiac investigation by the cardiologist. We attempted to minimise this bias by including seven different cardiologists, and the consistency between regression models of ACI and ADACI suggest appropriate selection of patients for additional cardiological intervention in this study. Finally, the categorical representation of risk factors such as hypertension, diabetes and hypercholesterolaemia does not distinguish between patients in whom these comorbidities are well controlled versus poorly controlled. Future studies should aim to collect objective parameters such as systolic blood pressure, blood sugar level and total cholesterol so as to improve assessment of the impact of these comorbidities.

## Clinical implications and future research

Despite these limitations, the predictive model presented here has broader implications for clinical practice which may be used to assist orthopaedic surgeons with more evidence-based referral to cardiology prior to TJA. The finding that a family history of cardiovascular disease had the greatest power in our model suggests the presence of this risk factor should prompt the surgeon to refer for pre-operative cardiac assessment. In the absence of FHx of CVD, male patients should also be referred given that a male of mean age and with a mean EF would reach the model cut-off score. This study also highlights the value of age and ejection fraction in determining which patients would benefit from pre-operative cardiology referral prior to undergoing TKA or THA. Additionally, the proposed model may serve as an example for future studies attempting to create a TJA-specific pre-operative cardiac referral tool, particularly for asymptomatic patients. Future investigations in this area should employ larger and more tailored patient cohorts that are specifically designed to develop a comprehensive tool that considers all risk factors associated with cardiac complication post-TJA that have been identified in the literature.

## Conclusions

As arthroplasty procedures increase exponentially, a validated pre-operative cardiac risk stratification tool is crucial to assist decision-making by orthopaedic surgeons with regard to patient referral for pre-operative cardiac optimisation. Increasing age, male gender, FHx of CVD, and reduced EF were identified as significant predictors for detection of cardiac abnormality in asymptomatic patients on pre-operative work-up. Future predictive models require larger cohorts which evaluate all potential risk factors of post-operative cardiac complication. Targeted pre-operative cardiac optimisation will not only reduce the risk of post-operative cardiac complication, but also has the potential to reduce health expenditure and surgical delays.

## Supplementary Information

**Additional file 1.** Table 1. Demographic characteristics and risk factors of patients in the total patient population. Supplementary Figure 1: Inclusion and exclusion criteria. TKA = total knee arthroplasty; THA = total hip arthroplasty; ECG = electrocardiogram; ECHO = echocardiogram. Supplementary Figure 2: A simplified flowchart of the analytical process. All data analysis used SPSS, V.24, P < 0.05 statistically significant. TKA = Total Knee Arthroplasty; THA = Total Hip Arthroplasty.

## Data Availability

The datasets used and analysed during the current study are available from the corresponding author on reasonable request.

## Abbreviations

ACI: Additional cardiac investigation
ADACI: Abnormality detected on additional cardiac investigation
ASA: American Society of Anesthesiologists
BMI: Body mass index
CAD: Coronary artery disease
CI: Confidence interval
CTCA: CT-coronary angiography
CVA: Cerebrovascular accident
CVD: Cardiovascular disease
ECG: Electrocardiogram
ECHO: Echocardiogram
EF: Ejection fraction
FHx: Family history
HTN: Hypertension
MIBI: Myocardial perfusion scan
NSQIP: National Surgical Quality Improvement Program
OR: Odds ratio
ROC: Receiver operator characteristics
THA: Total hip arthroplasty
TJA: Total joint arthroplasty
TKA: Total knee arthroplasty
