# Supplementary material for: Pre-operative clinical predictors for cardiology referral prior to total joint arthroplasty: the ‘asymptomatic’ patient
Source: J Orthop Surg Res. 2020 Nov 10;15:513. doi: 10.1186/s13018-020-02042-5 (PMC7654604; doi:10.1186/s13018-020-02042-5)

Additional Table 1 Demographic characteristics and risk factors of patients in the total patient population.

| **Continuous Variables** | **Mean** ± **SD (range)** | |
| --- | --- | --- |
| Age (years) | 69.94 ± 9.02 (44 – 94) | |
| BMI | 31.24 ± 5.35 (17.40 – 46.90) | |
| Ejection Fraction (%) | 63.82 ± 8.28 (23 – 81.00) | |
| **Categorical Variables** | **No. of patients (% of study cohort)** | |
|  | **Yes** | **No** |
| TKA | 272 (72.7%) | 102 (27.3%) |
| THA | 102 (27.3%) | 272 (72.7%) |
| Male | 202 (54.0%) | 172 (46.0%) |
| Female | 172 (46.0%) | 202 (54.0%) |
| Diabetes | 56 (15.0%) | 318 (85.0%) |
| Hypertension | 244 (65.2%) | 130 (34.8%) |
| Hypercholesterolaemia | 216 (57.8%) | 158 (42.2%) |
| **Smoking History** |  |  |
| Current | 22 (5.9%) |  |
| Ex-Smoker | 91 (24.3%) |  |
| Non-smoker | 261 (69.8%) |  |
| Excess alcohol history | 42 (11.2%) | 332 (88.8%) |
| Cardiac History | 132 (35.3%) | 242 (64.7%) |
| PVD | 7 (1.9%) | 367 (98.1%) |
| CVA | 27 (7.2%) | 347 (92.8%) |
| VTE | 14 (3.7%) | 360 (96.3%) |
| CKD | 23 (6.1%) | 348 (93.0%) |
| Asthma | 44 (11.8%) | 330 (88.2%) |
| COPD | 14 (3.7%) | 360 (96.3%) |
| OSA | 43 (11.5%) | 331 (88.5%) |
| Family history of CVD | 72 (19.3%) | 302 (80.7%) |
| ECG abnormality | 115 (30.7%) | 259 (69.3%) |

Numerical variables are reported as a mean ± standard deviation (SD) whilst categorical variables are reported as frequencies: number of patients (%). BMI = body mass index; TKA = total knee arthroplasty; THA = total hip arthroplasty; PVD = peripheral vascular disease; CVA = cerebrovascular disease; VTE = venous thromboembolism; CKD = chronic kidney disease; COPD = chronic obstructive pulmonary disease; OSA = obstructive sleep apnoea; CVD = cardiovascular; ECG = electrocardiogram.


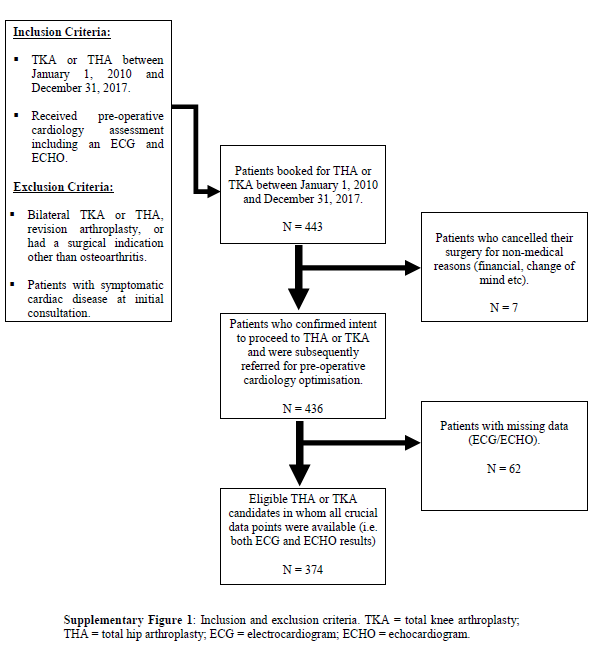


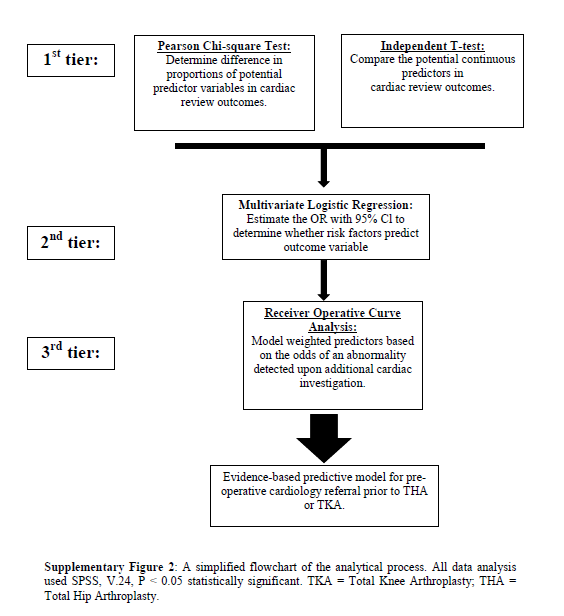

Supplement: Supplementary file 1 — Additional file 1. Table 1. Demographic characteristics and risk factors of patients in the total patient population. Supplementary Figure 1: Inclusion and exclusion criteria. TKA = total knee arthroplasty; THA = total hip arthroplasty; ECG = electrocardiogram; ECHO = echocardiogram. Supplementary Figure 2: A simplified flowchart of the analytical process. All data analysis used SPSS, V.24, P < 0.05 statistically significant. TKA = Total Knee Arthroplasty; THA = Total Hip Arthroplasty. [file 13018_2020_2042_MOESM1_ESM.docx]
